# Trajectories for the evolution of bacterial CO_2_-concentrating mechanisms

**DOI:** 10.1073/pnas.2210539119

**Published:** 2022-12-01

**Authors:** Avi I. Flamholz, Eli Dugan, Justin Panich, John J. Desmarais, Luke M. Oltrogge, Woodward W. Fischer, Steven W. Singer, David F. Savage

**Affiliations:** ^a^Department of Molecular and Cell Biology, University of California, Berkeley, CA 94720; ^b^Division of Biology and Biological Engineering, California Institute of Technology, Pasadena, CA 91125; ^c^Resnick Sustainability Institute, California Institute of Technology, Pasadena, CA 91125; ^d^Biological Systems and Engineering Division, Lawrence Berkeley National Laboratory, Berkeley, CA 94720; ^e^Division of Geological & Planetary Sciences, California Institute of Technology, Pasadena, CA 91125; ^f^HHMI, Chevy Chase, MD 20815; ^g^University of California, Berkeley, CA 94720

**Keywords:** carbon fixation, evolution, photosynthesis, Earth history, synthetic biology

## Abstract

Cyanobacteria rely on CO_2_-concentrating mechanisms (CCMs) to grow in today’s atmosphere (0.04% CO_2_). These complex physiological adaptations require ≈15 genes to produce two types of protein complexes: inorganic carbon (Ci) transporters and 100+ nm carboxysome compartments that encapsulate rubisco with a carbonic anhydrase (CA) enzyme. Mutations disrupting any of these genes prohibit growth in ambient air. If any plausible ancestral form—i.e., lacking a single gene—cannot grow, how did the CCM evolve? Here, we test the hypothesis that evolution of the bacterial CCM was “catalyzed” by historically high CO_2_ levels that decreased over geologic time. Using an *E. coli* reconstitution of a bacterial CCM, we constructed strains lacking one or more CCM components and evaluated their growth across CO_2_ concentrations. We expected these experiments to demonstrate the importance of the carboxysome. Instead, we found that partial CCMs expressing CA or Ci uptake genes grew better than controls in intermediate CO_2_ levels (≈1%) and observed similar phenotypes in two autotrophic bacteria, *Halothiobacillus neapolitanus* and *Cupriavidus necator*. To understand how CA and Ci uptake improve growth, we model autotrophy as colimited by CO_2_ and HCO_3_^−^, as both are required to produce biomass. Our experiments and model delineated a viable trajectory for CCM evolution where decreasing atmospheric CO_2_ induces an HCO_3_^−^ deficiency that is alleviated by acquisition of CA or Ci uptake, thereby enabling the emergence of a modern CCM. This work underscores the importance of considering physiology and environmental context when studying the evolution of biological complexity.

## Significance

The emergence of biological novelty is often coupled to the evolution of Earth’s chemical environment. Here, we studied how the evolution of a bacterial CO_2_-concentrating mechanism (CCM)—a complex, multicomponent system that enables modern CO_2_-fixing bacteria to grow robustly in environments with low CO_2_—depends on environmental CO_2_ levels. Using a “synthetic biological” approach to assay the growth of the present-day bacteria engineered to resemble ancient ones, we show that it is possible to explain the emergence of bacterial CCMs if atmospheric CO_2_ was once much higher than today, consistent with geochemical proxies. Taken together, our results delineated an unexpected “CO_2_-catalyzed” pathway for the evolution of bacterial CCMs, whose multiple emergence has been challenging to understand.

Nearly all carbon enters the biosphere through CO_2_ fixation in the Calvin–Benson–Bassham (CBB) cycle. Rubisco, the carboxylating enzyme of that pathway, is often considered inefficient due to relatively slow carboxylation kinetics (1–3) and nonspecific oxygenation of its five-carbon substrate ribulose 1,5-bisphosphate, or RuBP (4, 5). However, rubisco arose more than 2.5 billion years ago, when the Earth’s atmosphere contained virtually no O_2_ and, many argue, far more CO_2_ than today (6, 7). Over geologic timescales, photosynthetic O_2_ production (6), organic carbon burial (8), and CO_2_-consuming silicate weathering reactions (9) caused a gradual increase in atmospheric levels of O_2_ (≈20% of 1 bar atmosphere today) and depletion of atmospheric CO_2_ to the present-day levels of a few hundred parts per million (≈280 ppm preindustrial, ≈420 ppm or ≈0.04% today). Historical CO_2_ levels are challenging to estimate, but are thought to have been substantially higher than today, perhaps as high as 0.1–1 bar early in Earth history (7). It is likely, therefore, that contemporary autotrophs grow on much lower levels of CO_2_ than their ancestors did.

**Fig. 1.**
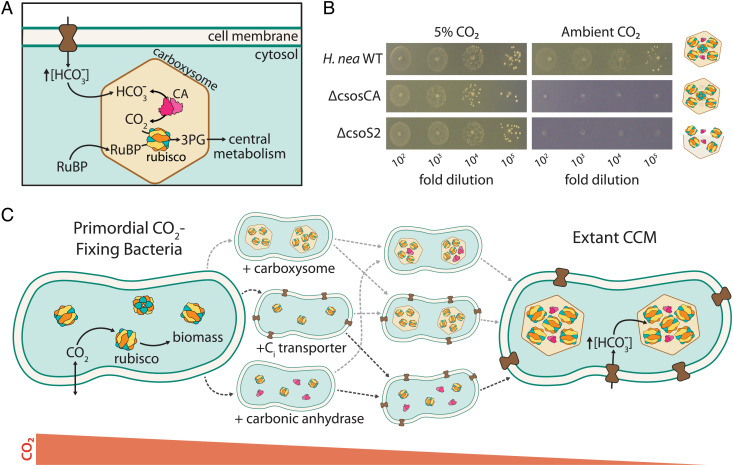
Mechanism and potential routes for the evolution of the bacterial CO_2_-concentrating mechanism. (*A*) Today, the bacterial CCM functions through the concerted action of three primary features - (i) an inorganic carbon (Ci) transporter at the cell membrane, and (ii) a properly-formed carboxysome structure (iii) co-encapsulating rubisco with carbonic anhydrase (CA). Ci uptake leads to a high intracellular HCO_3_^−^ concentration, well above equilibrium with the external environment. Elevated HCO_3_^−^ is converted to a high carboxysomal CO_2_ concentration by CA activity located only there, which promotes carboxylation by rubisco. (*B*) Mutants lacking genes coding for essential CCM components grow in elevated CO_2_ but fail to grow in ambient air, as shown here for mutations to the α-carboxysome in the proteobacterial chemoautotroph *H. neapolitanus*. Strains lacking the carboxysomal CA (Δ*csosCA*) or an unstructured protein required for carboxysome formation (Δ*csos2*) failed to grow in ambient air, but grew robustly in 5% CO_2_ (>10^8^ colony-forming units/ml, *SI Appendix*, Fig. S1). See *SI Appendix*, Table S4 for description of mutant strains. (*C*) We consider the CCM to be composed of three functionalities beyond rubisco itself: a CA enzyme (magenta), a Ci transporter (dark brown), and carboxysome encapsulation of rubisco with CA (light brown). If CO_2_ levels were sufficiently high, primordial CO_2_-fixing bacteria would not have needed a CCM. We sought to discriminate experimentally between the six sequential trajectories (dashed arrows) in which CCM components could have been acquired.

Many CO_2_-fixing organisms evolved CO_2_-concentrating mechanisms (CCMs), which help meet the challenge of fixing carbon in a low CO_2_ atmosphere. CCMs concentrate CO_2_ near rubisco and are found in several varieties in all Cyanobacteria, some Proteobacteria, as well as many eukaryotic algae and diverse plants (10). Because CO_2_ and O_2_ addition occur at the same active site in rubisco (4), elevated CO_2_ has the dual effects of accelerating carboxylation and suppressing oxygenation of RuBP by competitive inhibition (10). As shown in Fig. 1*A*, bacterial CCMs are encoded by ≈15 genes comprising three primary features: Zi) an energy-coupled inorganic carbon (Ci) transporter at the cell membrane and ii) a cytosolic 100+ nm protein compartment called the carboxysome that iii) coencapsulates rubisco with a carbonic anhydrase (CA) enzyme (2, 11). Energized Ci transport produces a high HCO_3_^−^ concentration in the cytosol (≈30 mM, Fig. 1*A*), which is converted into a high carboxysomal CO_2_ concentration by CA activity, localized exclusively to the carboxysome (12, 13). While HCO_3_^-^ undergoes spontaneous dehydration to CO_2_, the uncatalyzed reaction is slow enough (≈10s equilibration times) that HCO_3_^-^ can enter the carboxysome diffusively, where it undergoes rapid CA-catalyzed dehydration (10). This description applies to both varieties of bacterial CCM: the α-carboxysome variety found in Proteobacteria and many Cyanobacteria, as well as the β form found exclusively in Cyanobacteria (11, 14, 15).

CCM genes are straightforward to identify experimentally as mutations disrupting essential CCM components prohibit growth in ambient air (Fig. 1*B*) and mutants are typically grown in 1% CO_2_ or more (16–20). At first glance, therefore, the CCM appears to be “irreducibly complex” as all plausible recent ancestors—e.g., strains lacking individual CCM genes—are not viable in the present-day atmosphere. Irreducible complexity is incompatible with evolution by natural selection, so we and others supposed that bacterial CCMs evolved over a protracted interval of Earth history when atmospheric CO_2_ concentrations were much greater than that today (10, 21–23). We therefore hypothesized that ancestral forms of the bacterial CCM (i.e., those lacking some genes and complexes required today) would have improved organismal growth in the elevated CO_2_ environments that prevailed when they arose.

To test the hypothesis, we constructed the present-day analogs of plausible CCM ancestors (henceforth “analogs of ancestral CCMs”) and tested their growth across a range of CO_2_ partial pressures. Note that we have not endeavored to generate precise reconstructions of ancient taxa—indeed, we lack sufficient information about the organisms and their environments to do so. Rather, we aimed to identify the key components that appear in all CCMs (10) and ask whether or not they improve growth on their own in some defined CO_2_ concentration. Our goal was to identify a stepwise pathway of gene acquisition supporting the evolutionary emergence of a bacterial CCM by improving growth in ever-decreasing CO_2_ concentrations (Fig. 1*C*). We focused on trajectories involving sequential acquisition of genetic components because CAs (24), Ci transporters (20), and homologs of carboxysome shell genes (14, 25) are widespread among bacteria and could therefore be acquired horizontally.

**Fig. 2.**
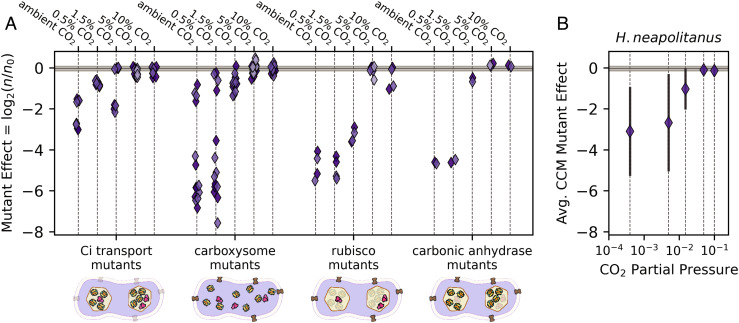
*H. neapolitanus* CCM genes contribute to growth even in super-ambient CO_2_ concentrations. *H. neapolitanus* is a chemoautotroph that natively utilizes a CCM in low CO_2_ environments. We profiled the contributions of CCM genes to autotrophic growth across a range of CO_2_ levels by assaying a barcoded transposon mutant library (20) in sequencing-based batch culture competition assays (*Methods*). Mutational effects were estimated as the log_2_ ratio of strain counts between the end point sample (e.g., 0.5% CO_2_) and the 5% CO_2_ preculture for each barcoded mutant (20, 28). As the library contained an average of ≈40 mutants per CCM gene, each point in (*A*) gives the average effect of multiple distinct mutants to a single CCM gene in a given CO_2_ condition. A value of log_2_(n/n_0_) = −2 therefore indicates that gene disruption was, on average, associated with a fourfold decrease in mutant abundance as measured by Illumina sequencing of mutant strain barcodes (20, 28). Negative values indicate that the gene, e.g., the carboxysomal CA, contributes positively to the growth of wild-type *H. neapolitanus* in the given condition. Observed log_2_(n/n_0_) values indicated that many *H. neapolitanus* CCM genes contribute to growth in super-ambient CO_2_ concentrations, including genes coding for Ci uptake, carboxysome shell proteins and the carboxysomal CA. Biological replicates are indicated by shading, and the gray bar gives the interquartile range of fitness effects for all ≈1700 mutants across all CO_2_ levels (−0.15-0.065). Replicates were highly concordant (*SI Appendix*, Fig. S2). “Ci transport mutants” include 4 DAB genes in two operons (20), “carboxysome” includes 6 nonenzymatic carboxysome genes, “rubisco mutants” denote the two subunits of the carboxysomal rubisco, and “CA mutants” denote the carboxysomal CA gene *csosCA*. *H. neapolitanus* also expresses a secondary rubisco, which explains why disruption of the carboxysomal rubisco is not lethal in high CO_2_ (*SI Appendix*, Fig. S3). Panel (*B*) gives the average mutational effect of CCM genes as a function of the CO_2_ concentration. Negative average values in 0.5-1.5% CO_2_ highlight the positive contribution of CCM genes to growth in these conditions. See *SI Appendix*, Figs. S2 and S3 for analysis of reproducibility and *SI Appendix*, Tables S1–S3 for detailed description of genes.

One approach to constructing contemporary analogs of CCM ancestors is to remove CCM genes from a native host. If CCM components were acquired sequentially, some single-gene knockouts would be analogous to recent ancestors, e.g., those lacking a complete carboxysome shell (26). We tested this approach by assaying a whole-genome knockout library of a ɣ-proteobacterial chemoautotroph, *H. neapolitanus,* in five CO_2_ partial pressures (20, 27). As shown in Fig. 2 and elaborated below, we found that many CCM genes contribute substantially to growth even at CO_2_ concentrations tenfold greater than the present-day atmosphere (0.5% CO_2_, ≈12.5 times the present atmospheric levels in 2020, PAL), supporting the view that CCM components play an important physiological role even in relatively high environmental CO_2_ concentrations.

Removing single CCM genes from a native host can only produce analogs of recent ancestors, however. We recently constructed a functional α-carboxysome CCM in an engineered *E. coli* strain called CCMB1 (2). This strain depends on rubisco carboxylation for growth and expression of a full complement of CCM genes from the chemoautotroph *H. neapolitanus* enabled growth in ambient air. Here, we used CCMB1 to construct analogs of ancestral CCMs, including several lacking one or more essential components of modern CCMs. We assayed the growth of these putative ancestors across a range of CO_2_ pressures to determine whether any ancestral forms contribute to organismal fitness—i.e., improve growth relative to a control strain expressing only rubisco—across a range of CO_2_ pressures. We had expected these experiments to highlight the central role of the carboxysome compartment (23), but instead found that CAs and/or Ci uptake systems were likely early drivers of CCM evolution. In the following sections, we describe these experiments, discuss how they highlight the central role of bicarbonate (HCO_3_^−^) in all metabolisms, and comment on how these results can inform our entwined understandings of bacterial physiology, CCM evolution, and the CO_2_ content of Earth’s ancient atmosphere.

## Results and Discussion

### *H. neapolitanus* CCM Genes Contribute to Fitness Even in Elevated CO_2_.

Using barcoded genome-wide transposon mutagenesis, we previously demonstrated that a 20-gene cluster in *H. neapolitanus* contains all the genes necessary for a functional CCM (2, 20). Our original screen measured the effect of gene disruption across the entire genome via batch competition assays, comparing the abundance of disruptive mutants in high CO_2_ (5%, ≈125 PAL) and ambient air (≈0.04%) via high-throughput sequencing (20, 28). If the relative abundance of mutants in a particular gene decreased reproducibly in ambient air, but not in 5% CO_2_, we concluded that the gene is linked to autotrophic growth in ambient air and, therefore, likely participates in the CCM. Our mutant “library” includes an average ≈35 distinct mutants per gene, so each “mutant fitness assay” contains multiple internal biological replicates.

To mimic the changes in atmospheric CO_2_ that likely occurred over Earth history, we assayed the same library in three additional CO_2_ pressures to cover five CO_2_ levels: ambient (≈0.04% CO_2_), low (0.5%, 12.5 PAL), moderate (1.5%, 37.5 PAL), high (5%, 125 PAL), and very high (10%, 250 PAL). Replicate experiments were strongly correlated (R > 0.85, *SI Appendix*, Fig. S2), implying a high degree of reproducibility. We, therefore, proceeded to ask whether *H. neapolitanus* CCM genes contribute to growth in a range of CO_2_ concentrations.

Fig. 2 plots the effect of disrupting CCM genes across five CO_2_ pressures, with genes grouped by their documented roles in the CCM. Each point in Fig. 2*A* represents the average fitness of 5–50 individual mutants. Surprisingly, we found that many CCM genes also contributed substantially to growth in 0.5% and 1.5% CO_2_, as indicated by large growth defects in disruptive mutants (negative values in Fig. 2*A*), resulting in a negative average impact of CCM mutants when the CO_2_ pressure was 1.5% or less (Fig. 2*B*). Carboxysome genes, for example, were critical for growth in 0.5% CO_2_, while certain Ci transport genes contributed substantially to growth in 0.5% and 1.5% CO_2_.

In high CO_2_, however, the *H. neapolitanus* CCM appears to be entirely dispensable (5–10%, Fig. 2*B*). As such, the data presented in Fig. 2 indicated that individual CCM components such as the carboxysome, CA, or Ci transporter may improve autotrophic growth in intermediate CO_2_ levels (≈1%) even in the absence of a complete and functional CCM. We considered testing this hypothesis in *H. neapolitanus* directly by constructing “ancestral-like” CCMs lacking carboxysome shell genes or Ci transporters. However, genetic manipulation of *H. neapolitanus* is cumbersome, and the native host’s regulatory network could complicate interpretation—a concern highlighted by three DNA-binding proteins that emerged in our screen as likely regulators of the CCM (*SI Appendix*, Fig. S3 and Table S1). We, therefore, decided to construct and test analogs of CCM ancestors in a nonnative host, namely *E. coli*.

### Evaluating Putative Ancestral CCMs in a Rubisco-Dependent *E. coli*.

**Fig. 3.**
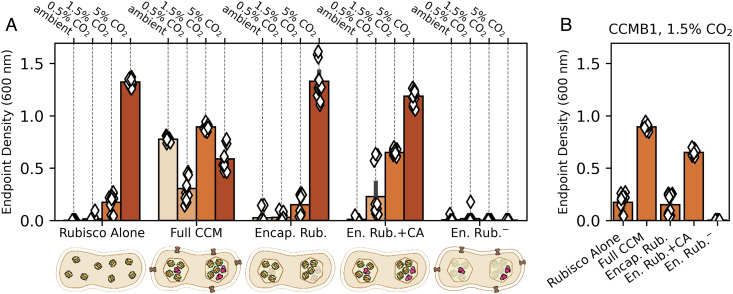
Expression of carboxysome genes without other CCM components does not improve the growth of an engineered rubisco-dependent *E. coli* in any CO_2_ level tested. We recently reconstituted a functional *H. neapolitanus* α-carboxysome CCM in a rubisco-dependent *E. coli* strain, CCMB1, by expressing 20 CCM genes from 2 plasmids (2). Here, we generated plasmid variants to test whether carboxysome expression improves rubisco-dependent growth in any of the four CO_2_ partial pressures during growth in a gas-controlled plate reader (*Methods*). Each diamond gives the end point optical density (600 nm) after 4 d of cultivation for one technical replicate of four biological replicates. The CCMB1 strain grows in elevated CO_2_ (1.5 and 5%) when rubisco is expressed (“Rubisco Alone”, *Left*). As previously reported, expressing the full complement of CCM genes from the pCB’ and pCCM’ plasmids (“Full CCM”) enabled growth in all CO_2_ levels. By replacing pCCM’ with a vector control and making an inactivating mutation to the carboxysomal CA (CsosCA C173S), we were able to express rubisco in a carboxysome without CA or Ci transport activities (“Encap. Rub.”). This strain grew similarly to the reference “Rubisco Alone” in all conditions. When the CA active site was left intact (“En. Rub.+CA”), growth improved above the “Rubisco Alone” baseline in 0.5% and 1.5% CO_2_. A negative control strain carrying inactive rubisco (“En. Rub.^−^”, CbbL K194M) failed to grow in all CO_2_ conditions. (*B*) Focusing on the growth in 1.5% CO_2_ highlights the contribution of CA activity to rubisco-dependent growth. See *SI Appendix*, Tables S4 and S5 for description of strains and plasmids, and see *SI Appendix*, Figs. S4 and S5 for growth curves and analysis of statistical significance.

We recently developed an *E. coli* strain, CCMB1, that depends on rubisco carboxylation for growth in minimal medium. This strain requires elevated CO_2_ for rubisco-dependent growth, but expressing the *H. neapolitanus* CCM from two plasmids enabled growth in ambient air (2). One of these plasmids, pCB’, expresses the carboxysome genes along with the encapsulated rubisco and CA enzymes (2). pCB’ derives from pHnCB10, which we routinely use to purify whole carboxysomes from *E. coli* (29, 30). The second plasmid, pCCM’, encodes the DAB1 Ci transporter, two rubisco chaperones, and a carboxysome positioning system (2, 20, 31, 32). We previously confirmed that CCM expression from these two plasmids enables growth in ambient air by i) verifying carboxysome formation by purification and electron microscopy, ii) demonstrating that several targeted mutations to CCM components abrogate growth in ambient air, and iii) measuring incorporation of isotopically labeled CO_2_ into biomass (2). Here, we used this two-plasmid system to express analogs of putative CCM ancestors in CCMB1 and assayed these strains’ growth in ambient air, 0.5%, 1.5%, and 5% CO_2_. We compared the growth of strains expressing partial CCMs to that of a reference CCMB1 strain expressing the *H. neapolitanus* Form IA rubisco on a vector, termed p1A, that expresses no other CCM components (2). As indicated by end point culture densities, the reference strain grew robustly in 5% CO_2_ but failed to grow in ambient air (Fig. 3*A*, “Rubisco Alone”). Full growth curves are given in *Supplementary Information*.

Consistent with our previous work, expression of the full complement of CCM genes permitted growth in all CO_2_ concentrations tested, as evident from the end point optical densities of these cultures (Fig. 3*A*, “Full CCM”). As the carboxysome is typically presented as the centerpiece of the bacterial CCM, we presumed that rubisco encapsulation played a pivotal role in CCM evolution. Indeed, recent modeling efforts support this hypothesis with calculations suggesting that encapsulation of rubisco in a semipermeable barrier could improve CO_2_ fixation by generating an acidic local pH (23). To evaluate the effect of encapsulating rubisco in a protein compartment, we replaced the pCCM’ plasmid with a vector control so that no Ci transporter was expressed and further deactivated the carboxysomal CA by mutating a single cysteine residue (pCB’ CsosCA C173S, “Encap. Rub.” in Fig. 3*A*). This pair of plasmids did not improve growth over the reference strain in any CO_2_ concentration tested. However, when we left the CA active site intact on otherwise identical plasmids (“En. Rub.+CA”), growth improved substantially in intermediate CO_2_ levels (0.5% and 1.5%). These data indicated that a carboxysomal CA plays a pivotal role in 0.5% and 1.5% CO_2_ (23), though it was unclear from these experiments whether carboxysome genes contribute to growth in these conditions.

### CA and Energy-Coupled Ci Transport Improve the Growth of Rubisco-Dependent *E. coli*.

Our observation that expression of the carboxysomal CA improves rubisco-dependent growth on its own (i.e., without also expressing a Ci transporter) motivated us to test the effects of CA and Ci transport independently of carboxysome expression. We designed plasmids that express the *H. neapolitanus* Dab2 Ci transporter (20), *E. coli*’s native CA (Can), or both. This was achieved by cloning both Dab2 and Can into a dual expression vector and making targeted active site mutants (DabA C539A, Can C48A, D50A) to isolate each activity. These vectors were cotransformed into CCMB1 with a constitutive version of p1A (p1Ac) so that rubisco expression would not be affected by induction of Dab2 or Can (*Methods*).

**Fig. 4.**
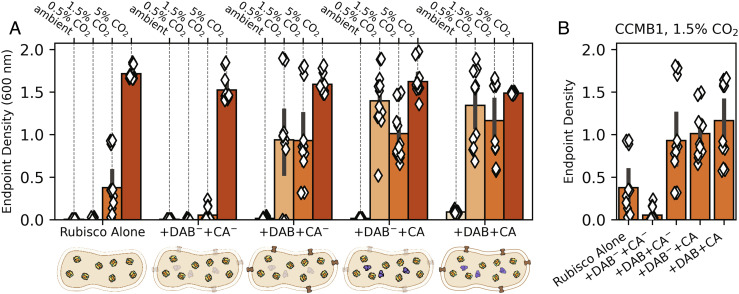
Expression of CA or Ci transport improves rubisco-dependent growth of CCMB1 *E. coli* in intermediate CO_2_ levels even in the absence of other CCM components. As shown in Fig. 3, CCMB1 *E. coli* strains were grown for 4 d in minimal medium in a gas-controlled plate reader. Each diamond gives the optical density (600 nm) after 4 d for one technical replicate of four biological replicates (*Methods*). The reference strain (“Rubisco Alone”) constitutively expresses rubisco from the p1Ac plasmid and carries a second plasmid, pFA-sfGFP, as a vector control. The reference grew only in 1.5% and 5% CO_2_. The remaining strains expressed both the *E. coli* native β CA (*can*) and the DAB2 Ci transporter from a pFA-family plasmid. These two activities were isolated by means of active site mutations. The negative control “+DAB^-^+CA^-^” expressed inactive Can (C48A, D50A) and DAB2 (DabA2 C539A) and grew less robustly than the reference in all conditions. If either active site was left intact (“+DAB+CA^-^” or “+DAB^-^+CA”), we observed a sizable growth improvement in both 0.5 and 1.5% CO_2_. Contrary to our expectations, this growth improvement remained when both active sites were left intact (“+DAB+CA”). Panel (*B*) emphasizes this effect by focusing on growth in 1.5% CO_2_. See *SI Appendix*, Table S4 for strain genotypes and *SI Appendix*, Figs. S6 and S7 for growth curves and analysis of statistical significance.

Expressing active Can and Dab2, whether alone or together, improved growth substantially in 1.5% CO_2_ (compare “+DAB+CA^−^,” “+DAB^−^+CA,” and “+DAB+CA” to “Rubisco Alone” in Fig. 4). This effect was even more pronounced in 0.5% CO_2_, where the reference strain failed to grow (Fig. 4*A* and *SI Appendix*, Fig. S7). Similar to the reference strain, a double-negative control strain expressing inactivated versions of both Dab2 and Can (“+Dab^−^+CA^−^”) grew poorly or not at all in 0.5% and 1.5% CO_2_, implying that the observed growth improvements were due to activity and not a side effect of heterologous gene expression.

It was not immediately obvious to us how CA and Ci uptake activities improve rubisco-dependent growth of CCMB1 *E. coli*. We found the effects of Dab2 expression especially perplexing because Ci uptake is expected to generate intracellular HCO_3_^−^ (11–13, 20, 33) while the rubisco substrate is CO_2_ (34). We observed similar phenotypes when expressing the cyanobacterial Na^+^:HCO_3_^−^ symporter, *sbtA* (11, 35), in CCMB1 (*SI Appendix*, Fig. S8). To confirm that these results are not a side effect of working in an engineered *E. coli* strain, but rather a genuine feature of autotrophy, we pursued experiments in a natively autotrophic proteobacterium, *C. necator*.

### *C. necator* Depends on CA for Autotrophic Growth.

While all photosynthetic Cyanobacteria rely on the CBB cycle and a full complement of CCM genes (14), some chemoautotrophic bacteria depend on the CBB cycle but lack identifiable genes encoding carboxysome components or Ci transporters (36, 37). As most characterized bacterial rubiscos are not CO_2_ saturated in ambient air and are, in addition, substantially inhibited by atmospheric levels of O_2_ (5), we expected that such organisms would require elevated CO_2_ for robust growth.

**Fig. 5.**
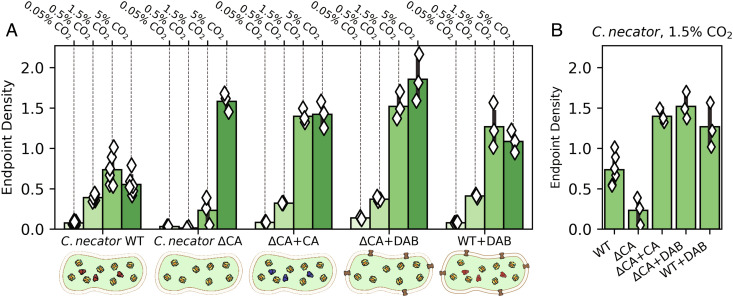
*C. necator* requires CA or Ci uptake for robust autotrophic growth in 0.5% and 1.5% CO_2_. *C. necator* strains were grown autotrophically in minimal medium at a variety of CO_2_ levels, and end point optical density was measured after 48 h (*Methods*). (*A*) Growth of the *C. necator* double CA knockout (ΔCA) was greatly impaired in 0.5% and 1.5% CO_2_. Compared to wild-type *C. necator* (WT), which grew to a final OD600 of 0.73 ± 0.28 in 1.5% CO_2_ (six biological replicates), growth of ΔCA was greatly impaired, reaching a final OD of 0.23 ± 0.17 (three biological replicates). Expression of either the human CA II (ΔCA+CA) or the DAB2 Ci transporter from *H. neapolitanus* (ΔCA+DAB) recovered robust growth which exceeded even the wild type, indicating that the wild type may not express saturating levels of CA. Panel (*B*) focuses on 1.5% CO_2_. See *SI Appendix*, Fig. S9 for statistical analysis.

*Cupriavidus necator*, formerly known as *Ralstonia eutropha*, is one such bacterium (38, 39). *C. necator* is a facultative chemolithoautotroph typically found at the interface between oxic and anoxic environments where H_2_ and O_2_ coexist. Such “knallgas” environments include soils, sediments, and geothermal sites (40) that are often characterized by elevated CO_2_ (40, 41). While *C. necator* is an obligate aerobe capable of chemoautotrophic growth on H_2_, CO_2_, and O_2_ via the CBB cycle, it has no carboxysome genes and no obvious Ci transporters (37). Consistent with previous reports (42), autotrophic growth of wild-type *C. necator* was very poor in ambient CO_2_ (“*C. necator* WT” in Fig. 5*A*). We generated a double CA knockout strain, *C. necator* Δ*can* Δ*caa* (43, 44), and found that CA removal greatly attenuated autotrophic growth in 0.5% and 1.5% CO_2_ (“*C. necator* ΔCA”). Consistent with our experiments in *E. coli*, this growth defect was complemented by heterologous expression of a human CA (“ΔCA+CA”) or a DAB-type Ci uptake system (“ΔCA+DAB”). Moreover, as in *E. coli*, coexpression of Ci uptake with native CAs was not deleterious (“WT+DAB”). Rather, this strain grew to higher densities than wild type in 1.5% and 5% CO_2_ (e.g., Fig. 5*B*).

### A Nutritional Requirement for HCO3- Explains the Observed Phenotypes.

**Fig. 6.**
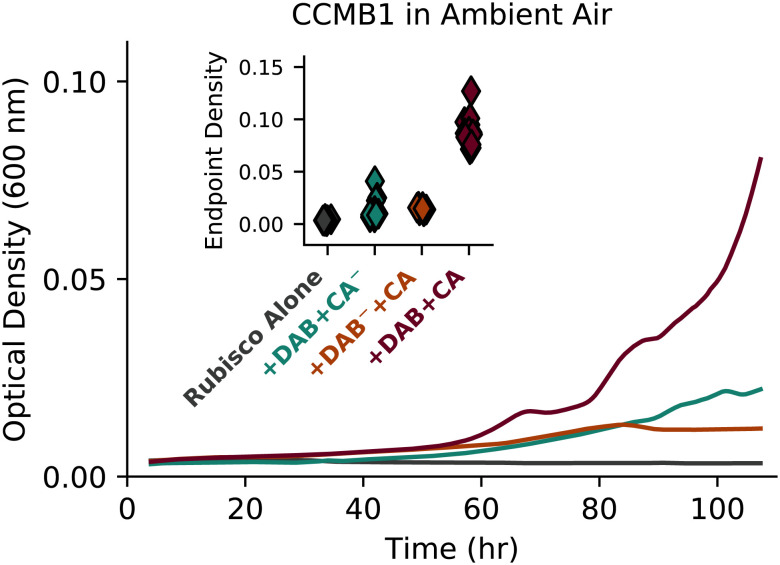
Coexpression of CA and Ci uptake enabled rubisco-dependent growth of CCMB1 *E. coli* in ambient air. Inspecting the ambient CO_2_ growth data presented in Fig. 4 revealed that coexpression of CA and Ci transport (“Rub.+DAB+CA”) substantially improved rubisco-dependent growth of CCMB1 *E. coli* in ambient CO_2_ concentrations. This effect was modest (≈0.1 OD units above the “Rubisco Alone” control) but reproducible, as indicated by end point data plotted on the inset. Curves are colored to match labels on the inset. See *SI Appendix*, Fig. S10 for statistical analysis.

We found that expression of a CA, Ci transporter, or both improved rubisco-dependent growth of *C. necator* and CCMB1 *E. coli* in intermediate CO_2_ concentrations (0.5 and 1.5%, Figs. 4 and 5). Furthermore, to our surprise, a CCMB1 strain expressing active Dab2 and Can grew reproducibly, if slowly, in ambient air (“+Dab+CA” in Fig. 6). This was remarkable because biological membranes are very permeable to CO_2_, with a permeability coefficient P_C_ ≈ 0.1-1 cm/s (13, 45, 46). Given this high permeability, we expected coexpression of energized HCO_3_^−^ uptake with a CA to generate a deleterious “futile cycle” where energy-expended pumping HCO_3_^−^ was wasted when CO_2_ produced by CA activity “leaks” back out of the cell. Indeed, landmark experiments in a model Cyanobacterium demonstrated that expressing a cytosolic CA alongside native Ci uptake greatly reduced intracellular Ci concentrations and inhibited photosynthesis (12). Here, we instead found that such a cycle is compatible with rubisco-dependent growth of two bacteria in relatively low CO_2_ (1.5% or lower).

We initially entertained a naive explanation for how CA expression might improve growth: that it increases the intracellular CO_2_ concentration relative to the reference strain expressing only rubisco. This could arise if rubisco activity depletes intracellular CO_2_ (C_in_) significantly below its extracellular level (C_out_) in the reference strain. In this setting, one might assume that CA activity accelerates the equilibration of CO_2_ across the membrane, thereby increasing C_in_ and carboxylation flux. However, this naive model cannot explain the growth benefits associated with expressing Ci uptake systems, which provide HCO_3_^−^ and not CO_2_ (11, 13, 32). Moreover, the following calculation shows that this hypothesis is unreasonable precisely because CO_2_ is so membrane permeable that rubisco cannot deplete C_in_ much beneath C_out_.

In a bacterium, rubisco might make up 20% of soluble protein at the very most (47). This amounts to a mass concentration of roughly 0.2 × 300 mg/ml ≈ 60 mg/ml rubisco (48). As each rubisco active site is attached to ≈60 kDa of protein (BNID 105007), the maximum rubisco active site concentration is ≈1 mM. In this naive model, C_in_ is set by the balance of passive uptake through the membrane, with effective permeability α = P_C_ × SA/V ≈ 10^4^ s^−1^, and fixation by rubisco, with an effective rate constant of γ = [rubisco] × k_cat_/K_M_ < 10^3^ s^−1^ (neglecting inhibition by O_2_). These values give a steady-state C_in_ of α C_out_ / (α + γ) > 0.9 C_out_. That is, even an extreme level of rubisco activity cannot draw C_in_ beneath 90% of C_out_. In such conditions, rubisco would fix ≈10^10^ CO_2_/hour, supporting a 1-2 h doubling time (see *SI Appendix* for full calculation). So, although the CO_2_ concentration is low in aqueous environments equilibrated with the present-day atmosphere (≈10–20 μM), passive CO_2_ uptake is expected to be very fast, giving intracellular CO_2_ concentrations nearly equal to extracellular ones and supporting substantial carboxylation flux. As C_in_ ≈ C_out_, CA activity cannot substantially affect C_in_ or carboxylation flux in this setting.

**Fig. 7.**
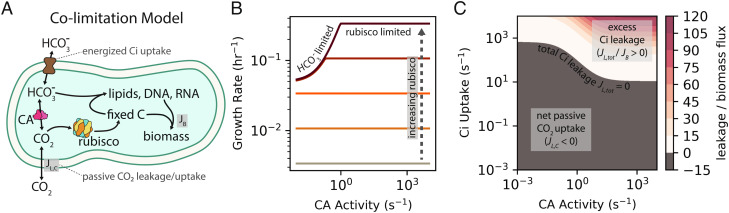
Colimitation of autotrophic growth by CO_2_− and HCO_3_^−^-dependent carboxylation reactions can explain the growth improvements associated with expressing CAs and Ci transporters. (*A*) In autotrophs using the CBB cycle, nearly all biomass carbon derives from rubisco-catalyzed CO_2_ fixation. However, autotrophs also require HCO_3_^−^ for carboxylation reactions in lipid, nucleic acid, and arginine biosynthesis (49–51). We expressed this diagram as a mathematical model, which we applied to understand why CA and Ci uptake improved rubisco-dependent growth. (*B*) The model exhibited two regimes: one wherein growth was limited by rubisco flux and another where it was limited by HCO_3_^−^-dependent carboxylation (“bicarboxylation”) flux. At low rubisco levels (lighter-colored lines), growth was rubisco limited: increased rubisco activity produced faster growth, but the growth rate was insensitive to CA activity because slow spontaneous CO_2_ hydration provided sufficient HCO_3_^−^ to keep pace with rubisco. At higher rubisco levels (maroon lines), growth was bicarboxylation limited and increased CA activity was required for increasing rubisco activity to translate into faster growth. Increasing Ci uptake led to similar effects (*SI Appendix*, Fig. S12). In panel (*C*), color indicates the ratio of total Ci leakage (*J_L,tot_* = *J_L,C_* + *J_L,H_*) to biomass production flux (*J_B_*) at fixed rubisco activity across wide ranges of CA and Ci uptake activities. *J_L,tot_* was calculated as the sum of CO_2_ and HCO_3_^−^ leakage rates (*J_L,C_* + *J_L,H_*) with *J_L,C_* ≫ *J_L,H_* in most conditions due to the much greater membrane permeability of CO_2_. The so-called futile cycling, where leakage greatly exceeds biomass production (*J_L,tot_* / *J_B_* ≫ 1), occurs when CA and Ci uptake are coexpressed at extreme levels (redder colors). See *SI Appendix* for detailed description of the colimitation model.

We instead argue that the effects of expressing CA or Ci transport can be explained by the ubiquitous dependence of growth on HCO_3_^−^ (49–52). It has been clear for at least 80 y that heterotrophs also require Ci for growth. Seminal investigations in the 1930s and 1940s advanced the hypothesis that this dependence is due to a specific requirement for HCO_3_^−^ (53–56), which is now known to be the substrate of several carboxylation reactions involved in lipid, nucleotide, and arginine biosynthesis (49, 50, 57, 58). This explanation is now supported by experiments in several heterotrophs demonstrating that growth in ambient air can be supported either by CA activity (49–51) or by providing the products of central metabolic carboxylations in the growth media (50, 51). Similar CA mutant phenotypes were recently observed in a model plant (52), and the metabolic networks of microbial autotrophs indicate an equivalent dependence on HCO_3_^−^ for anabolism (59, 60). We, therefore, advance a model of colimitation of autotrophic growth by both CO_2_− and HCO_3_^−^-dependent carboxylation fluxes, where most of the biomass carbon derives from rubisco-catalyzed carboxylation of CO_2_ and a small minority derives from HCO_3_^−^ (Fig. 7*A*). We formalized this notion as a simplified linear differential equation model fully described in *SI Appendix*. This model intentionally omits several facets of autotrophic physiology for the sake of simplicity (e.g., enzyme saturation, rubisco oxygenation) and is therefore designed for coarse, order-of-magnitude comparisons with data, not direct quantitative correspondence.

We first used our model to confirm that rubisco should not be expected to deplete intracellular CO_2_. Only when rubisco activity was very high or the modeled CO_2_ permeability was implausibly low could the intracellular CO_2_ (C_in_) be depleted beneath 50% of the extracellular level (C_out_). Furthermore, when C_in_ ≈ C_out_, neither CA activity nor Ci uptake meaningfully increased the modeled rubisco carboxylation flux (*SI Appendix*, Fig. S13). When the HCO_3_^−^ dependence of biomass precursor production was included in our model, however, it became possible to rationalize the growth improvements associated with heterologous expression of a CA or Ci transporter, as both of these activities can supply HCO_3_^-^ needed for anabolic carboxylations (Fig. 7*A*). When the modeled rubisco activity was low (lighter colored lines in Fig. 7*B*), growth was rubisco-limited: increasing rubisco activity (moving toward darker colors in Fig. 7*B*) or the CO_2_ concentration (*SI Appendix*, Fig. S12*C*) produced faster growth, but the growth rate was insensitive to CA activity because slow CO_2_ hydration already provided sufficient HCO_3_^−^ for the anabolic carboxylations involved in lipid, nucleotide, and amino acid biosynthesis (*SI Appendix*, Figs. S12–S13). When rubisco activity was set to higher values (darker lines in Fig. 7*B*), the model entered a “bicarboxylation-limited” regime where increased rubisco activity did not affect the growth rate until CA activity was increased to supply HCO_3_^−^ (i.e., bicarbonate) for anabolic carboxylations. In bicarboxylation-limited conditions, additional HCO_3_^−^ could be supplied by CA, Ci uptake, or both (Fig. S12). However, these activities are not equivalent, as we discuss below.

The colimitation model also gave insight into the nature of the supposed “futile cycle,” which could now be framed quantitatively by comparing the leakage of Ci from the cell (flux *J_L,tot_* = *J_L,C_* + *J_L,H_*) to biomass production by rubisco- and HCO_3_^−^-dependent carboxylases (*J_B_*, Fig. 7*A*). As CO_2_ is orders of magnitude more membrane permeable than HCO_3_^−^ near neutral pH (13), CO_2_ leakage (*J_L,C_*) greatly exceeded the HCO_3_^−^ leakage flux (*J_L,H_*) in most modeled conditions. Low Ci leakage is desirable; when *J_L,tot_* ≈ 0, the flux of Ci pumped into the cell is balanced by fixation of CO_2_ and HCO_3_^−^ such that no energy is “wasted” on Ci uptake. When CA (δ) and Ci uptake (χ) activities were low, the model predicted slow growth and net diffusive Ci uptake (*J_L,C_*, *J_L,H_* < 0) to balance carboxylation (Fig. 7*C* and *SI Appendix*, Fig. S15). Increasing Ci uptake (χ) could cause *J_L,tot_* to change sign from negative (connoting passive uptake) to positive (connoting leakage) passing through *J_L,tot_* = 0. If CA was also expressed—i.e., if δ was increased above a baseline value—*J_L,tot_* ≈ 0 could be achieved at lower Ci uptake activities due to CA-catalyzed dehydration of imported HCO_3_^−^ producing CO_2_ used by rubisco. In other words, CA activity lowers the Ci uptake rate and, consequently, energy expenditure required to achieve the same rate of biomass production (Fig. 7*C* and *SI Appendix*, Fig. S15). When both activities were high, the model predicted substantial leakage, with *J_L,tot_* / *J_B_* ≈ 100 in extreme cases. Such extreme levels of futile cycling are likely incompatible with growth. If Ci uptake was low, in contrast, CA activity could be increased arbitrarily without incurring leakage because CAs do not pump Ci into the cell (Fig. 7*C*).

A second, less plausible way in which coexpression might improve growth is if CA and Ci transport are fast and membrane permeability to CO_2_ is far lower than typically assumed. In this case, the combination of Ci uptake and CA activity might form a CO_2_ pump that elevates C_in_ substantially above C_out_ to accelerate rubisco carboxylation (*SI Appendix*, Fig. S14). As HCO_3_^−^ is supplied sufficiently by Ci uptake in this case, the colimitation model predicted that increased rubisco fluxes would translate into faster growth. For this effect to arise, however, the membrane permeability to CO_2_ would have to be 100–1,000 times lower than the measured (45) or calculated (46), so CO_2_ pumping is unlikely to explain the capacity of a CCMB1 to grow in ambient air when coexpressing Dab2 and Can (Fig. 6).

Taken together, our experiments and model helped outline a plausible trajectory for the coevolution of the bacterial CCM with atmospheric CO_2_ levels. Presuming an ancestral autotroph with only a rubisco-driven CBB cycle and no CCM components, our data and model support a trajectory where CA and Ci transport are acquired together or serially (in either order) to support growth as atmospheric CO_2_ levels decreased (darker arrows in Fig. 1*C*). The order of acquisition might depend on the environmental pH, which strongly affects the extracellular HCO_3_^−^ concentration and, thereby, the expected efficacy of HCO_3_^−^ uptake (13). The colimitation model helped us understand the potential advantages of expressing CA and Ci uptake together: modest coexpression can reduce energy expended on Ci pumping and balance the supply of CO_2_ and HCO_3_^−^ with the cellular demand for rubisco and bicarboxylation products (Fig. 7*C* and *SI Appendix*, Fig. S15). In CCMB1, we found that the coexpression of CA and Ci transport supported growth in low CO_2_ environments (≈1%, Fig. 4) and even permitted modest growth in atmosphere (Fig. 6); cells expressing both activities would have been “primed” for the subsequent acquisition and refinement of proto-carboxysome structures that coencapsulate rubisco and CA to enable robust growth at yet lower CO_2_ levels (e.g., ambient air, Fig. 3). Notably, carboxysome shell proteins are structurally related to two ubiquitous protein families (25) and homologous to other metabolic microcompartments (14, 25), suggesting two plausible routes for the acquisition of carboxysome genes.

Results from our *E. coli* experiments suggest that these evolutionary trajectories are “fitness positive” in that each step improves growth as environmental CO_2_ levels decrease (Figs. 3, 4, and 6). Moreover, the contribution of CA and Ci transport activities was only realized at intermediate pCO_2_ ≈ 1% in both *E. coli* (Fig. 4) and the chemoautotroph *C. necator* (Fig. 5), supporting the view that CO_2_ concentrations declining from high levels earlier in Earth history promoted the evolutionary emergence of bacterial CCMs (10).

## Concluding Remarks

There is great and longstanding interest in characterizing the composition of the atmosphere over Earth history (7, 8, 26, 61–63). This interest is surely justified as the contents of the atmosphere affected the temperature, climate, and the chemical conditions in which life arose, evolved, and has been maintained. In the case of reactive species like O_2_, the present-day measurements of old sedimentary rocks are quite informative: proxies for O_2_ reactivity in geological samples demonstrate that the ancient atmosphere contained very little O_2_, with inferred levels of 1 ppm or less in the Archean Eon, 4–2.5 billion years ago (6, 7). The emergence of oxygenic photosynthesis in Cyanobacteria led to the “Great Oxidation Event” ≈2.5 billion years ago (64) and atmospheric O_2_ levels have increased in a punctuated fashion since (65). Geochemical models and proxies generally agree that the ancient atmosphere was also CO_2_ rich, with atmospheric CO_2_ levels generally declining over the last 3 billion years due to geochemical cycles and organic carbon burial (7, 8,26, 63).

Here, we used the tools of synthetic biology to ask whether the history of atmospheric CO_2_ helps resolve the apparent “irreducible complexity” of the bacterial CCM, which enables autotrophic growth in the present-day atmosphere (0.04% CO_2_, 21% O_2_) only when all genetic components are intact (Fig. 1*B*). Irreducible complexity is incompatible with evolution by natural selection, and so we hypothesized that partial CCMs conferred a growth advantage in the historical environments in which they arose, which we presume were characterized by higher levels of CO_2_ than found in today’s atmosphere (7, 63).

Results from our experiments with rubisco-dependent *E. coli* and two bacterial autotrophs—*H. neapolitanus* and *C. necator*—supported this rationalization of CCM evolution by showing i) that CO_2_ ≈ 1% (25 PAL) improves the growth of organisms harboring partial CCMs (Figs. 3–6) and ii) that all CCM genes are dispensable during growth in 5-10% CO_2_ (Figs. 2–5). This latter result suggested to us that atmospheric CO_2_ concentrations exceeded 1% of a 1 bar atmosphere when rubisco arose, which was likely more than 3 billion years ago (21, 66). If geological CO_2_ sinks later brought atmospheric CO_2_ to ≈1%, all organisms, including autotrophs, would have begun to evolve or acquire CAs and/or Ci transporters to provide the HCO_3_^−^ required for biosynthesis (Fig. 7). An ancestral autotroph expressing both of these activities may have had a growth advantage in relatively lower CO_2_ pressures < 1% (Fig. 6) and would have been “primed” for the evolution of a CCM as the only missing component, the carboxysome, could have evolved from oligomeric host proteins (25) or may have been adapted from a different metabolic microcompartment (14). Alternatively, it is possible that CAs (or Ci transporters) arose prior to rubisco and were already widespread at the time of rubisco evolution, in which case we might expect CO_2_ ≳ 1% when rubisco arose. Unfortunately, the convergent evolution of CA activity in several protein families (67) makes it very challenging to constrain the timing of CA evolution with comparative biological and molecular clock approaches; this issue concerns bacterial Ci transporters as well (11, 20).

While we focused here on understanding the relationship between atmospheric CO_2_ and the emergence of bacterial CCMs, O_2_ is a competitive inhibitor of rubisco carboxylation (4, 5) and atmospheric O_2_ levels have increased dramatically over Earth history (68). A full description of CCM evolution should therefore account for atmospheric O_2_ as well as CO_2_. However, many CO_2_-fixing organisms, including Cyanobacteria, algae, plants, and all those studied here, often utilize O_2_ in their metabolisms. CCMB1 *E. coli* respires glycerol (2), while *H. neapolitanus* uses O_2_ as the preferred terminal electron acceptor during chemoautotrophic growth (69), as does *C. necator* (37). Since O_2_ is used for fundamental bioenergetic needs, the O_2_ dependence of growth cannot be used as a proxy for the effect of O_2_ on the CCM, at least for the bacteria studied here.

It is intrinsically difficult to answer questions about Earth’s deep biological history; addressing such questions will surely require cooperation between scientific disciplines. Here, we took a “synthetic biological” approach to study the molecular evolution of bacterial CO_2_ fixation by constructing contemporary cells intended to resemble ancient ones in certain ways (26, 66, 70). Our work highlighted the impact of environmental context (CO_2_ concentrations) and whole cell physiology (the requirement for HCO_3_^−^) on the evolution of CO_2_ fixation. That is, neither rubisco nor the CCM should be considered in isolation, but rather in the context of a metabolism that demands both CO_2_ and HCO_3_^−^ for growth. We hope that future research advances the synthetic approach to studying evolution and fully expect that this approach will enrich our understanding of biological processes that have shaped the evolution of biogeochemical cycles on Earth.

## Methods

### Strains, Plasmids, and Genomic Modifications.

Strains and plasmids used in this study are documented in *SI Appendix*, Tables S4 and S5. The rubisco-dependent *E. coli* strain CCMB1 was derived from *E. coli* BW25113 and has the genotype BW25113 Δ*rpiAB* Δ*edd* Δ*cynT* Δ*can*, as documented in ref. 2. To construct CA-deficient mutants of *C. necator* H16, we first knocked out the hdsR homolog *A0006* as removal of this restriction enzyme increases electroporation efficiency (37). The CA knockout, *C. necator* H16 Δ*A0006* Δ*can* Δ*caa*, was constructed by repeated rounds of selection and counter selection by integrating a construct encoding both kanamycin resistance and *sacB* for counter selection. Protocols for plasmid construction and genomic modification of *C. necator* are fully described in the *SI Appendix*.

### Genome-Wide Fitness Measurements in *H. neapolitanus*.

Competitive fitness assays were performed following (20). The barcoded *H. neapolitanus* transposon library generated for that work was thawed and used to inoculate three 33 ml cultures that were grown overnight at 30 C in DSMZ-68 (pH 6.8) with 10 μg/ml kanamycin. These precultures were grown to an OD ≈ 0.07 (600 nm) in 5% CO_2_. The library was subsequently back diluted 1:64 and grown in various CO_2_ concentrations (10%, 5%, 1.5%, 0.5%, and ambient CO_2_) on a platform shaker (New Brunswick Scientific Innova 2000, 250 RPM) in a Percival Intellus Incubator configured to mix pure CO_2_ with laboratory air to reach the desired CO_2_ partial pressure; 20 ml of preculture was pelleted by centrifugation (15 min at 4,000 g) and saved as a T_0_ reference. Upon reaching 6.5–7.5 doublings, 50 ml of culture was spun down and gDNA was extracted for barcode PCRs as described in ref. 6; barcodes were previously mapped to the genome via TnSeq (20). PCRs were purified (Zymo Research Clean and Concentrator kit) and pooled for sequencing on an Illumina MiSeq with 150 bp single-end reads. We used the software pipeline from ref. 28 to analyze barcode abundance data. Briefly, fitness of individual mutant strains was calculated as the log_2_ of the ratio of barcode abundance in the experimental condition over abundance in the T_0_. Gene-level fitness values were then calculated considering all transposon insertions expected to disrupt an individual gene. Each CO_2_ concentration was tested in biological duplicate except for 5% CO_2_, which was assayed in biological quadruplicate.

### *E. coli* Growth Conditions.

*E. coli* strains were grown at 37°C. 60 μg/ml kanamycin and 25 μg/ml chloramphenicol were used for selection during routine cloning and propagation. For strains carrying two selectable markers, antibiotics were used at half concentration (30 μg/ml kanamycin, 12.5 μg/ml chloramphenicol). All CCMB1 cultures were grown in 10% CO_2_ unless otherwise specified. When rubisco-independent growth was desired, CCMB1 was propagated in rich LB media. CCMB1 was cultured in a rubisco-dependent manner in M9 minimal media (pH 7.0) supplemented with trace elements and 0.4% glycerol (v/v) as described in ref. 2. Experiments presented in Figs. 3, 4, and 6 were conducted in 96-well plates in a gas-controlled plate reader (Tecan Spark) configured to mix pure CO_2_ with laboratory air to reach a defined CO_2_ partial pressure. For the data in Fig. 3, 100 nM anhydrous tetracycline (aTc) was supplied to induce expression from p1A, pCB’, and pCCM’ plasmids. For the data presented in Figs. 4 and 6, a constitutive version of p1A was used (p1Ac) and aTc was omitted from precultures. These strains also carried a dual-expression plasmid, pFC, for inducible expression of the DAB2 Ci transport operon and the *can* CA. 100 nM aTc was supplied to induce DAB2 and 1 mM IPTG for *can*.

Precultures were inoculated into 5 ml of M9 glycerol with 30 μg/ml kanamycin, 12.5 μg/ml chloramphenicol, and appropriate induction. 1 ml of each culture was transferred to a separate tube, which was incubated in ambient CO_2_ as a negative control; the remaining 4 ml was incubated in 10% CO_2_. CCMB1 strains carrying active site mutants of rubisco (cbbL K194M) were precultured in LB with the same antibiotic concentrations. Once cultures reached saturation, they were centrifuged at 4000 g for 8 min. Pellets were washed in 10 ml of M9 with no carbon source (M9 NoC) and resuspended in 5 ml M9 NoC. Optical density was measured in fivefold dilution at 600 nm (Genesys 20 spectrophotometer, Thermo Scientific), and cultures were normalized to 0.5 OD units. 96-well plates were inoculated by adding 2.5 μl of OD-normalized preculture to 247.5 μl M9 glycerol supplemented with appropriate antibiotics and induction to a starting density of 0.005 OD600. Kanamycin was omitted as the plasmid carrying kanamycin resistance also expresses rubisco, which is required for growth in glycerol media (2). To minimize evaporation during multiday cultivations, 150 μl sterile water was added to the reservoirs between the wells and the plate was incubated inside of a small humidity cassette (Tecan) with 3 ml sterile water added to each reservoir. The plates were incubated with shaking for at least 4 d in a Tecan Spark plate reader configured to control the CO_2_ concentration and measure the culture density (OD600) every 30 min. The humidity cassette was replenished after 48 h. All experiments were performed in biological quadruplicate and technical triplicate.

### *C. necator* Growth Conditions.

*C. necator* strains were grown in ambient CO_2_ unless otherwise specified, and 200 μg/ml kanamycin was added to select for plasmid retention. When rubisco-independent heterotrophic growth was desired, *C. necator* was cultured in LB at 30°C. For autotrophic growth experiments, strains were precultured in 5 mL of LB media in 10% CO_2_ in 25 mL tubes with 20 mm butyl stoppers sealed by aluminum crimping. Precultures were incubated for 2 d at 30°C with 200 rpm shaking, washed three times in *Cupriavidus* minimal media (pH 6.7), and inoculated to an OD of 0.1 (550 nm) in minimal media in a 165-mL flask with a 20-mm butyl stopper sealed by aluminum crimping. *Cupriavidus* minimal growth media contained 3.24 mM MgSO_4_, 0.42 mM CaCl_2_, 33.5 mM NaH_2_PO_4_, 32.25 mM Na_2_HPO_4_, 2.6 mM K_2_SO_4_, 1 mM NaOH, 1.87 mM NH_4_Cl, and 1 mL/L of a 1000× trace mineral solution containing 480 mg/L CuSO_4_·5H_2_O, 2.4 g/L ZnSO_4_·7H_2_O, 2.4 g/L MnSO_4_·H_2_O, and 15 g/L FeSO_4_·7H_2_O (pH 6.7). The flasks were evacuated, and the headspace was filled with 60% H_2_, 10% O_2_, and indicated concentrations of CO_2_. The balance was air. Cells were grown for 48 h at 30°C with 200 rpm shaking, and OD_550_ values were taken using a Molecular Devices SpectraMax M2 spectrophotometer.

## Supplementary Material

Appendix 01 (PDF)

Dataset S01 (XLSX)

## Data Availability

Heterogeneous data on microbial growth data have been deposited in [GitHub] (https://github.com/flamholz/ccm_evolution). Previously published data were used for this work (https://doi.org/10.1038/s41564-019-0520-8).
